# First description of *Echinococcus ortleppi* infection in China

**DOI:** 10.1186/s13071-019-3653-y

**Published:** 2019-08-09

**Authors:** Yunliang Shi, Xiaoling Wan, Ziyue Wang, Jun Li, Zhihua Jiang, Yichao Yang

**Affiliations:** 1 Institute of Parasitic Disease Prevention and Control, Guangxi Zhuang Autonomous Region Center for Disease Control and Prevention, Nanning, 530028 China; 2 School of Public Health, Guangxi Medical University, Nanning, 530021 China

**Keywords:** *Echinococcus ortleppi*, Hydatid, Human, China

## Abstract

**Background:**

Echinococcosis has led to considerable social and economic losses in China, particularly in the endemic communities of the eastern Tibetan Plateau. In China, human cases of *Echinococcus granulosus* (*sensu stricto*), *E. canadensis* and *E. multilocularis* infections have been described, but no *E. ortleppi* (G5) infections in humans or animals have been reported.

**Results:**

A case of *E. ortleppi* infection in a human from Guangxi, which is a non-endemic echinococcosis area in China, is described. A 17 × 12 × 20 cm (diameter) cyst was observed in the liver of the patient, and *Echinococcus* larvae were collected from the cyst. A morphological examination indicated that the larvae were *E. ortleppi*, and amplification and analysis of the nicotinamide adenine dinucleotide hydrogenase dehydrogenase subunit 1 (*nad*1) and cytochrome *c* oxidase subunit 1 (*cox*1) genes showed that the larvae had 99–100% homology with the corresponding *E. ortleppi* sequences on GenBank.

**Conclusions:**

To our knowledge, this report describes the first identification of a human *E. ortleppi* infection in China. Our data broaden the geographical distribution of this rarely reported species of *Echinococcus*.

**Electronic supplementary material:**

The online version of this article (10.1186/s13071-019-3653-y) contains supplementary material, which is available to authorized users.

## Background

Cystic echinococcosis is a globally distributed zoonotic disease caused by the larval form (metacestode) of the dog tapeworm genus *Echinococcus* (Cestoda: Taeniidae). Humans become infected through the ingestion of *Echinococcus* spp. eggs that have been deposited from the faeces of the definitive hosts (canids), and the larval stage develops in the viscera of humans [1]. Nine valid species of the genus *Echinococcus*: *E. granulosus* (*sensu stricto*) [G1 (sheep strain), G2 (Tasmanian sheep strain) and G3 (buffalo strain)]; *E. equinus* (G4); *E. ortleppi* (G5); *E. canadensis* (G6–G10); *E. vogeli*; *E. felidis*; *E. oligarthrus*; *E. multilocularis*; and *E. shiquicus*, have been identified [2–5]. The first five species have often been grouped under the common name *E. granulosus* (*sensu lato*)*. Echinococcus granulosus* (*s.s.*) and *E. multilocularis* are the two most important species responsible for human cystic echinococcosis and alveolar echinococcosis.

*Echinococcus ortleppi* was formerly known as the cattle strain of *E. granulosus* (*s.l.*) or genotype G5 [6, 7], and its morphology and developmental features show substantial differences compared with those of *E. granulosus* (*s.s.*) and other taxa. In addition to cattle, buffaloes, camels, sheep, pigs, goats, monkeys and deer can also be infected with *E. ortleppi* [8–12]. In recent years, several cases of *E. ortleppi* infection have been reported in Europe, South America, Africa and Asia [13]. Humans acquire the infection and become aberrant intermediate hosts through the accidental ingestion of parasite eggs and metacestodes develop in the liver or lungs.

Four species in the genus *Echinococcus* have been reported in China: *E. granulosus* (*s.s.*); *E. canadensis*; *E. multilocularis*; and *E. shiquicus* [14–19]. *Echinococcus granulosus* (*s.l.*) and *E. multilocularis* are the two common species, and infection with these species can lead to death if left untreated. Two *E. canadensis* (G7 and G10) human infection cases have been identified in Heilongjiang Province [17, 18], and *E. shiquicus* was isolated from a Tibetan fox in Shiqu County in the Qinghai-Tibet Plateau in 2005 [19]. To date, five genotypes of *E. granulosus* (*s.l.*), i.e. G1, G3, G6, G7 and G10, have been reported in China [14–18]. All five genotypes can infect humans, and the G1 and G6 genotypes can infect animals; however, the G1 genotype is predominant. Here, we report an *E. ortleppi* case in a human from Guangxi, South China, that was free of local hydatid infection.

## Methods

### Case presentation

The 65-year-old male described in this report was residing in Rongshui County, Liuzhou City, Guangxi Province, Southern China. At the time of study he was a teacher, was living in his place of birth, had never left the country prior to diagnosis and had not travelled to any echinococcosis- or schistosomiasis-endemic areas. In May 2017, he felt pain in the right upper abdomen; this pain was persistent and not accompanied by any paroxysmal aggravation, and all other clinical signs were not significant. He did not present with a fever, cough or other symptoms. He visited the People’s Hospital of Miao Autonomous County in Rongshui County, and his clinical diagnosis and abdominal ultrasonography indicated a “hepatic cyst”.

### Morphological and molecular identification

The surgery was conducted laparoscopically, and *Echinococcus* was found in and collected from the fluid-filled cyst in the liver. The morphology of the collected *Echinococcus* was assessed. The total DNA of the *Echinococcus* parasite was extracted using a commercial DNeasy® Blood & Tissue Kit (Qiagen, Hilden, Germany) in accordance with the manufacturer’s instructions. Two mitochondrial genes, cytochrome *c* oxidase subunit 1 (*cox*1) and nicotinamide dehydrogenase subunit 1 (*nad*1), were amplified using the primers F/COI (5′-TTG AAT TTG CCA CGT TTG AA TGC-3′) and R/COI (5′-GAA CCT AAC GAC ATA ACA TAA TGA-3′) [20], and JB11 (5′-AGA TTC GTA AGG GGC CTA ATA-3′) and JB12 (5′-ACCACTAACTAATTCACTTTC-3′) [21], respectively. The PCRs were performed in a reaction volume of 25 μl containing 4 μl of DNA, 12.5 μl of PCR mix (Takara, Dalian, China), 1 μl of each primer (10 pmol/μl) and 6.5 μl of PCR-grade water. The amplification consisted of 94 °C for 3 min followed by 35 cycles of denaturation at 94 °C for 1 min, annealing at 50 °C (*cox*1)/54 °C (*nad*1) for 1 min and extension at 72 °C for 1 min, and a final extension step at 72 °C for 10 min. The PCR products were analysed by electrophoresis in 1.5% agarose gels. The amplified DNA was purified from the gel using an Agarose Gel DNA Extraction Kit (Takara), cloned into a PMD-19T vector (Takara) and transformed and propagated into DH5α-competent *E. coli* (Takara).

The T plasmid vector-conserved primer M13 was used to select the positive clones, and the plasmid DNA was isolated from single colonies using a MiniBEST Plasmid Purification Kit (Takara). The recombinant plasmids were sequenced at Sangon Biotech (Shanghai, China). The sequences were compared with those available in the GenBank database using the Basic Local Alignment Search Tool (BLASTn; http://blast.ncbi.nlm.nih.gov/Blast.cgi). The phylogenetic trees of the *nad*1 and *cox*1 gene sequences of *E. ortleppi* China and other related strains were constructed using the maximum likelihood (ML) method with MEGA v.7.0 [22].

## Results

**Fig. 1:**
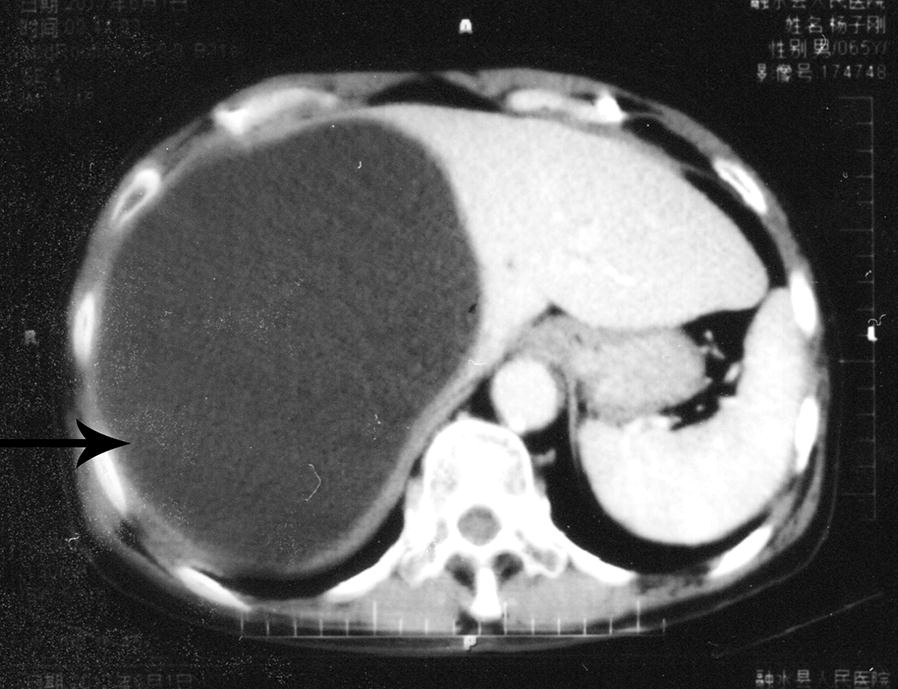
Abdominal ultrasonography detected a hydatid cyst (arrow) with a size of 17 × 12 × 20 cm (diameter) in the liver of the patient

**Fig. 2:**
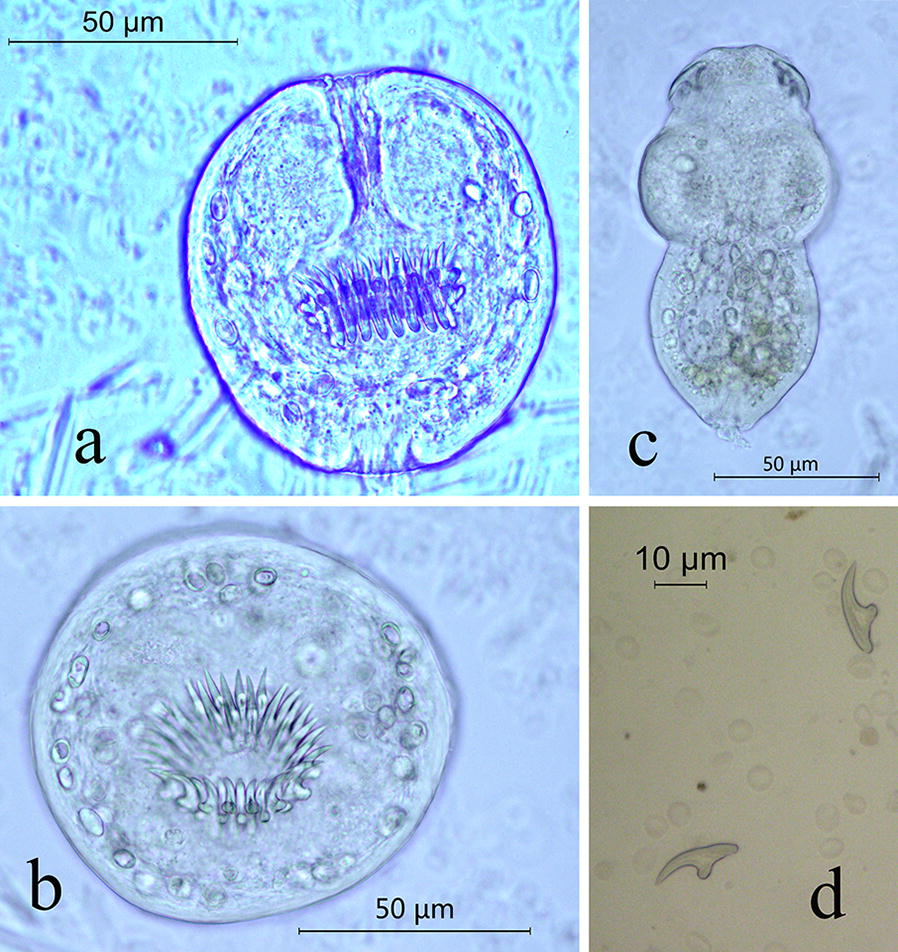
Protoscoleces and hooklets of *Echinococcus ortleppi.* **a** Invaginated scolices observed by microscopy (40×). **b** Evaginated scolices (40×). **c** Evaginated scolices of the protoscolices (40×). **d** Hooks of the larvae (40×)

Abdominal ultrasonography showed cystic-like low-density shadows in the right lobe of the liver; the boundary was clear, and the size of the shadow was approximately 17 × 12 × 20 cm (Fig. 1). No dilatation of the intrahepatic and extrahepatic bile ducts was observed, and no abnormal tissue was detected in the hilum of the liver. The size and shape of the gall-bladder and spleen were normal. According to the WHO ultrasound image classification guidelines [23], the CE type belongs to CE1-large (CE1l). A laparoscopic examination revealed compensatory enlargement of the left lobe of the liver, enlargement of the right lobe of the liver and morphological abnormalities. The cystic mass was approximately 17 × 12 × 20 cm in size and exhibited a clear cystic fluid, calcification of the cyst wall, and a layer of translucent white powdery material with a thickness of approximately 0.2–0.3 cm in the cyst. The cyst (cystic echinococcosis) contained many protoscoleces, each of which had many hooks (Fig. 2a–c). Each protoscolex appeared as a spherical body with a diameter of approximately 95 × 80 µm and an invaginated scolex with 30 hooks (Fig. 2b), and each hook was 20 μm (Fig. 2d).

A 935-bp nucleotide sequence for *cox*1 loci and a 530-bp sequence for *nad*1 loci were obtained through sequencing. The *cox*1 and *nad*1 sequences were submitted to the GenBank database under the accession numbers MN058591 and MN058592, respectively. The BLAST analysis showed that the *cox*1 sequence exhibited 99–100% homology with that of *E. ortleppi* in GenBank isolates previously collected in France (GenBank: KU743919, KU743922 and KU743922), Estonia (GenBank: KY766908, KY766906 and KY766907), Japan (GenBank: AB235846), Africa (e.g. GenBank: KU842045) and the UK (GenBank: KU378107). The *nad*1 sequences showed 99–100% homology to the *E. ortleppi* sequences in GenBank obtained from Estonia (GenBank: KY766908 and KY766907) and Sudan (GenBank: KU842045). Both *cox*1 and *nad*1 sequences showed only 93–94% homology to those of *E. granulosus*. The phylogenetic tree of *E. ortleppi* and other related strains derived from part of the *cox*1 and *nad*1 genes showed that the *E. ortleppi* strain collected in China belongs to the same group as the strains of *E. ortleppi* in GenBank (Additional file 1: Figure S1 and Additional file 2: Figure S2, respectively).

*Echinococcus ortleppi*-infected serum was detected using an ELISA kit (Combined Biotech Co. Ltd., Shenzhen, China) that could detect a wide range of *Echinococcus* [including *E. granulosus* (*s.s.*) and *E. multilocularis*] IgG antibodies and showed positive reactivity with *E. ortleppi*. To determine whether the animals and humans in the patient’s residential area were infected with *E. ortleppi*, 10 dog faeces samples were collected and examined using the Kato-Katz technique. Forty human serum samples from families, neighbours and colleagues of patients were collected and analysed using an ELISA kit (Combined Biotech Co. Ltd., Shenzhen, China) to detect *Echinococcus* IgG antibodies. No positive samples were detected from dogs or humans.

## Discussion

*Echinococcus ortleppi* is a cattle strain (genotype G5) of *E. granulosus* that is mainly transmitted between dogs and cattle, and humans become infected through the accidental ingestion of parasite eggs. To date, 11 cases of human infection have been reported in the Netherlands, Argentina, Mexico, South Africa, Brazil, France, India and Vietnam (Table 1) [24–32], and animal infections have been detected in cattle, monkeys, camels, pigs, cows, goats, sheep, oryx, bovines, crested porcupines and spotted deer in Asia (India, Vietnam, Egypt, Bhutan and Iran), Africa (Kenya, South Africa, Sudan, Ethiopia, Zambia and Namibia), South America (Brazil and Chile) and Europe (the UK, France and Italy) (Table 2) [3, 8–12, 24, 26, 33–42]. Human infection is rare, and it appears that human *E. ortleppi* infection is very uncommon and restricted to certain areas. *Echinococcus ortleppi* can cause high infection rates in cattle; in Brazil, the ratio of *E. granulosus* (*G1*) to *E. ortleppi* (*G5*) cysts in cattle is almost 1 to 1 [38].

**Table 1:** Summary of human cases of *Echinococcus ortleppi*

Year	Country	No. of cases	Cyst localization	Reference
1988	Netherlands	1	Spleen	Bowles et al. [24]
2002	Argentina	1	Liver	Kamenetzky et al. [25]
2004	Mexico	1	Liver	Maravilla et al. [26]
2010–2012	South Africa	1	Liver	Mogoye et al. [27]
2011	Brazil	1	–	de la Rue et al. [28]
2011–2012	France	2	Livers	Grenouillet et al. [29]
2013	India	1	Liver	Sharma et al. [30]
2017	Vietnam	2	Lungs	Van De & Le Van [31]
2018	France	1	Vertebral	Basmaciyan et al. [32]

**Table 2:** Summary of animal infection of *Echinococcus ortleppi*

Year	Country	Species	No. infected	Reference
Asia				
2009	India	Cattle/buffalo/pig	3/2/4	Pednekar et al. [8]
2009	Vietnam	Monkey	1	Pednekar et al. [8]
2015	Egypt	Camel	1	Amer et al. [11]
2016	Bhutan	Cattle	1	Thapa et al. [33]
2017	Iran	Camel	1	Ebrahimipour et al. [34]
Africa				
2004	Kenya	Cattle/pig	2/1	Dinkel et al. [9]
2010–2012	South Africa	Cow	1	Mogoye et al. [27]
2013	Sudan	Camel	1	Ahmed et al. [35]
2013	Kenya	Cattle/goat/sheep	23/3/2	Mbaya et al. [10]
2015	Ethiopia	Cattle/pig	5/1	Tigre et al. [36]
2016	Kenya	Cattle/goat/camel/sheep	54/3/2/1	Addy et al. [3]
2016	Zambia	Cattle/pig	52/1	Addy et al. [3]
2016	Namibia	Cattle/oryx	35/3	Addy et al. [3]
2016	Ethiopia	Cattle	7	Addy et al. [3]
2016	Sudan	Cattle/camel	15/1	Addy et al. [3]
2018	Kenya	Dog	1	Mulinge et al. [37]
America				
2002	Argentina	Cattle/dog	5/2	Kamenetzky et al. [25]
2012	Brazil	Cattle	277	Balbinotti et al. [38]
2016	Brazil	Cattle	7	Addy et al. [3]
2016	Brazil	Bovine	250	Monteiro et al. [39]
2018	Brazil	Crested porcupine	1	Hodzic et al. [40]
2018	Chile	Cattle	2	Correa et al. [41]
Europe				
2012	UK	Spotted deer	1	Boufana et al. [12]
2014	France	Cattle	7	Grenouillet et al. [29]
2016	France	Cattle	7	Addy et al. [3]
2008	Italy	Cattle	1	Casulli et al. [42]

China is one of the most important endemic regions of echinococcosis [43], and echinococcosis is a major parasitic problem in humans and livestock. It has been estimated that China has 0.6–1.3 million cases of human echinococcosis by 2000 [44]. Most of the provinces have reported human infections, and western and northwest China are the main endemic areas. The eastern Tibetan Plateau, China, has the highest reported prevalence of echinococcosis in the world [45]. *Echinococcus granulosus* and *E. multilocularis* are common species in China, and five genotypes of *E. granulosus* (*s.l.*), namely G1, G3, G6, G7 and G10, have been reported [14–18]. Here, we report a case of *E. ortleppi* (G5) infection in a human residing in a non-endemic echinococcosis area in China. It is generally believed that humans who are infected with echinococcosis in non-endemic areas obtained the infection mainly from endemic areas when they visited these areas or ingested the eggs on contaminated fruits from endemic areas. In this study, we did not detect hydatid infection in dog or human samples collected near the patient’s home. Because the incubation period of echinococcosis can be as long as 30 years, it is impossible to determine whether the reported case should be considered an imported or local case.

Three confirmed human cases of hydatid infections in Guangxi have been reported, and the patients did not reside in echinococcosis-endemic areas. Because the parasites were not subjected to molecular analysis, their species and genotype were not identified. Some echinococcosis cases in non-endemic areas of China have been reported, and the infection source in some of these cases has not been identified. We hypothesize that these patients might also have been considered cases of local *E. ortleppi* infection. Therefore, we strongly suggest that the parasites from patients with echinococcosis residing in non-endemic areas should be subjected to genotyping and molecular analysis to identify the *Echinococcus* species.

## Conclusions

This study reports a case of *E. ortleppi* infection that caused cystic echinococcosis in the liver of a Chinese patient. To our knowledge, this case constitutes the first detection of this species in China, and the identification of this first case highlights the need to enhance national surveillance efforts, particularly human, livestock and dog surveillance in non-endemic areas. Moreover, the *Echinococcus* species and genotype of infections should be detected.

## Additional files

**Additional file 1: Figure S1.** Phylogenetic tree for *Echinococcus* spp. based on the *cox*1 gene including the sequence of *E. ortleppi* from China.

**Additional file 2: Figure S2.** Phylogenetic tree for *Echinococcus* spp. based on the *nad*1 gene including the sequence of *E. ortleppi* from China.

## Data Availability

The data used in this study are available upon request from the corresponding author.

## Abbreviations

*cox*1: cytochrome *c* oxidase subunit 1
*nad*1: dehydrogenase subunit 1
