# Correction to: First description of *Echinococcus ortleppi* infection in China

**DOI:** 10.1186/s13071-019-3677-3

**Published:** 2019-08-23

**Authors:** Yunliang Shi, Xiaoling Wan, Ziyue Wang, Jun Li, Zhihua Jiang, Yichao Yang

**Affiliations:** 1 Institute of Parasitic Disease Prevention and Control, Guangxi Zhuang Autonomous Region Center for Disease Control and Prevention, Nanning, 530028 China; 2 School of Public Health, Guangxi Medical University, Nanning, 530021 China

## Correction to: Parasites Vectors (2019) 12:398 10.1186/s13071-019-3653-y

Following publication of the original article [1], the authors reported an error in Table 2.

Namely, in the ‘America’ section of the table the species ‘Crested Porcupine’ is matched with the country ‘Brazil’.

However, it should be matched with ‘Bosnia and Herzegovina’ (and so be placed in the ‘Europe’ section).

The corrected Table 2 is given in this erratum.

**Table 2:** Summary of animal infection of *Echinococcus ortleppi*

Year	Country	Species	No. infected	Reference
Asia				
2009	India	Cattle/buffalo/pig	3/2/4	Pednekar et al. [8]
2009	Vietnam	Monkey	1	Pednekar et al. [8]
2015	Egypt	Camel	1	Amer et al. [11]
2016	Bhutan	Cattle	1	Thapa et al. [33]
2017	Iran	Camel	1	Ebrahimipour et al. [34]
Africa				
2004	Kenya	Cattle/pig	2/1	Dinkel et al. [9]
2010–2012	South Africa	Cow	1	Mogoye et al. [27]
2013	Sudan	Camel	1	Ahmed et al. [35]
2013	Kenya	Cattle/goat/sheep	23/3/2	Mbaya et al. [10]
2015	Ethiopia	Cattle/pig	5/1	Tigre et al. [36]
2016	Kenya	Cattle/goat/camel/sheep	54/3/2/1	Addy et al. [3]
2016	Zambia	Cattle/pig	52/1	Addy et al. [3]
2016	Namibia	Cattle/oryx	35/3	Addy et al. [3]
2016	Ethiopia	Cattle	7	Addy et al. [3]
2016	Sudan	Cattle/camel	15/1	Addy et al. [3]
2018	Kenya	Dog	1	Mulinge et al. [37]
America				
2002	Argentina	Cattle/dog	5/2	Kamenetzky et al. [25]
2012	Brazil	Cattle	277	Balbinotti et al. [38]
2016	Brazil	Cattle	7	Addy et al. [3]
2016	Brazil	Bovine	250	Monteiro et al. [39]
2018	Chile	Cattle	2	Correa et al. [41]
Europe				
2012	UK	Spotted deer	1	Boufana et al. [12]
2014	France	Cattle	7	Grenouillet et al. [29]
2016	France	Cattle	7	Addy et al. [3]
2018	Bosnia and Herzegovina	Crested porcupine	1	Hodzic et al. [40]
2008	Italy	Cattle	1	Casulli et al. [42]
