# Supplementary figures and images for: First description of Echinococcus ortleppi infection in China
Source: Parasit Vectors. 2019 Aug 9;12:398. doi: 10.1186/s13071-019-3653-y (PMC6689172; doi:10.1186/s13071-019-3653-y)

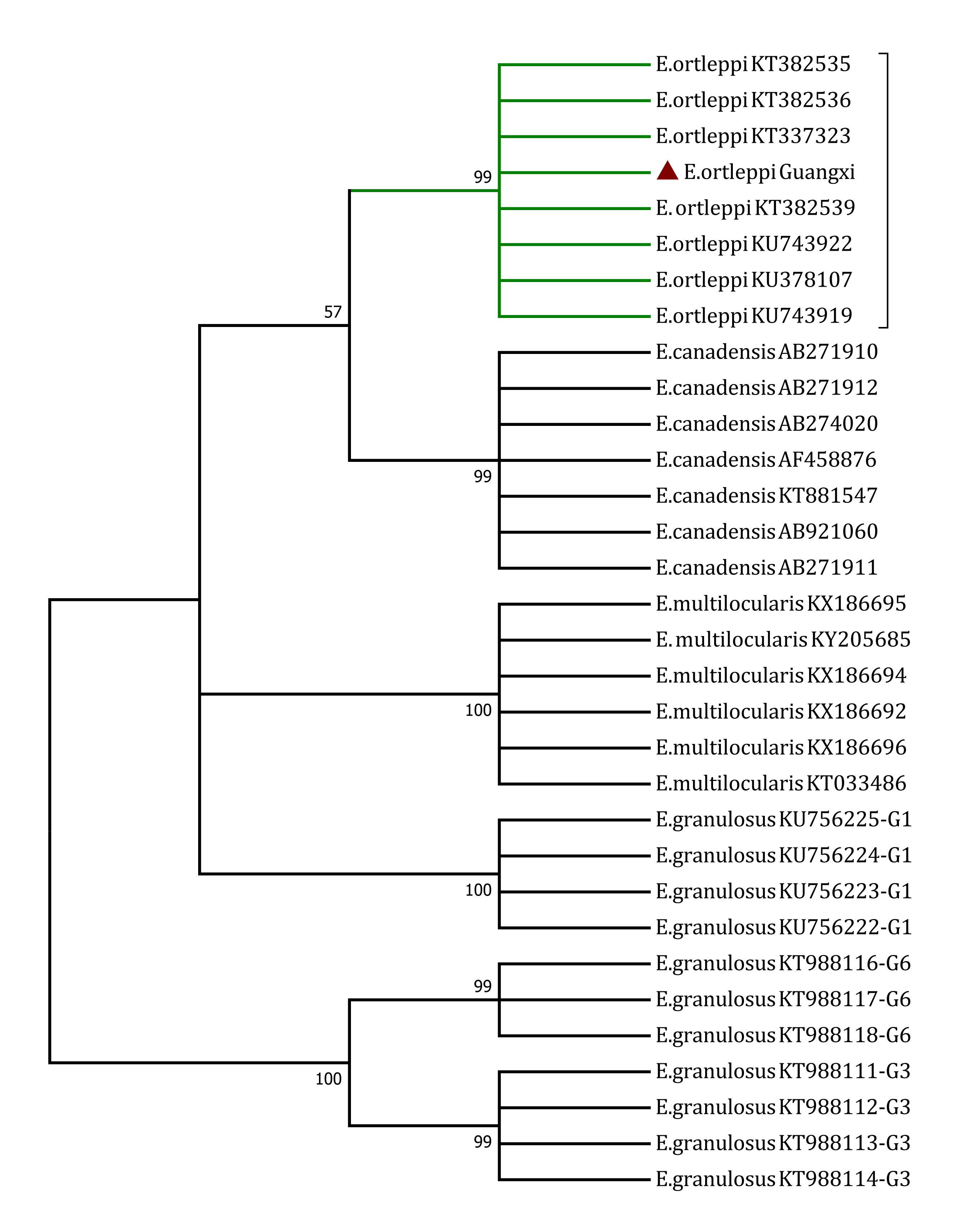

Supplement: Supplementary file 1 — Additional file 1: Figure S1. Phylogenetic tree for Echinococcus spp. based on the cox1 gene including the sequence of E. ortleppi from China. [file 13071_2019_3653_MOESM1_ESM.jpg]

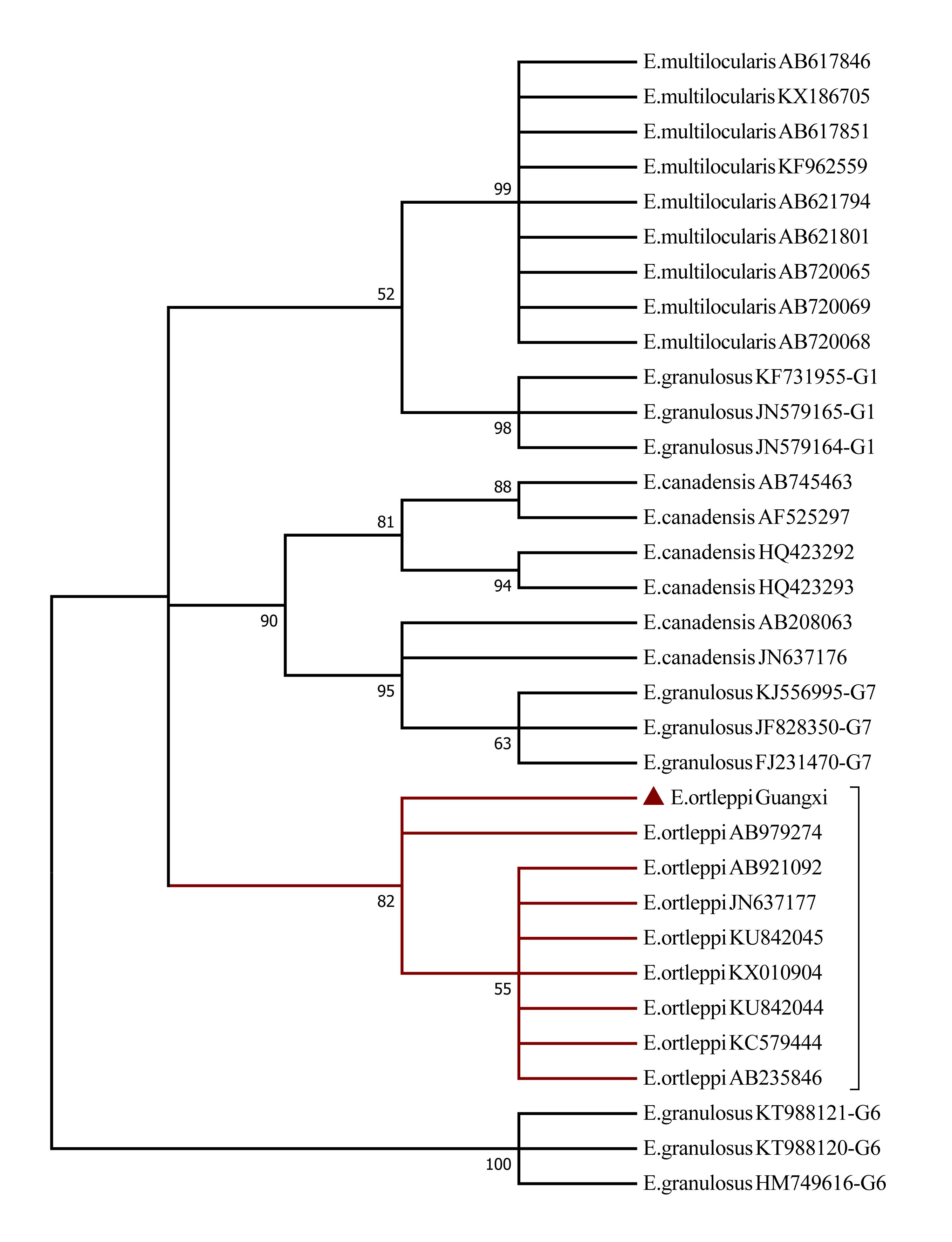

Supplement: Supplementary file 2 — Additional file 2: Figure S2. Phylogenetic tree for Echinococcus spp. based on the nad1 gene including the sequence of E. ortleppi from China. [file 13071_2019_3653_MOESM2_ESM.jpg]
